# The crest phenotype in domestic chicken is caused by a 197 bp duplication in the intron of *HOXC10*

**DOI:** 10.1093/g3journal/jkaa048

**Published:** 2021-01-04

**Authors:** Jingyi Li, Mi-Ok Lee, Brian W Davis, Ping Wu, Shu-Man Hsieh Li, Cheng-Ming Chuong, Leif Andersson

**Affiliations:** 1 Department of Veterinary Integrative Biosciences, College of Veterinary Medicine and Biomedical Sciences, Texas A&M University, College Station, TX 77843, USA; 2 Key Laboratory of Agricultural Animal Genetics, Breeding and Reproduction of Ministry of Education, College of Animal Science and Technology, Huazhong Agricultural University, 430070 Wuhan, Hubei, China; 3 Department of Pathology, University of Southern California, Los Angeles, CA 90033, USA; 4 Center for Craniofacial Molecular Biology, Ostrow School of Dentistry, University of Southern California, Los Angeles, CA 90033, USA; 5 Department of Biochemistry, National Defense Medical Center, Taipei 114, Taiwan; 6 Science for Life Laboratory, Department of Medical Biochemistry and Microbiology, Uppsala University, SE-751 23 Uppsala, Sweden; 7 Department of Animal Breeding and Genetics, Swedish University of Agricultural Sciences, SE-750 07 Uppsala, Sweden

**Keywords:** crest, skin regional specificity, cerebral hernia, IBD mapping, HOXC gene cluster

## Abstract

The *Crest* mutation in chicken shows incomplete dominance and causes a spectacular phenotype in which the small feathers normally present on the head are replaced by much larger feathers normally present only in dorsal skin. Using whole-genome sequencing, we show that the crest phenotype is caused by a 197 bp duplication of an evolutionarily conserved sequence located in the intron of *HOXC10* on chromosome 33. A diagnostic test showed that the duplication was present in all 54 crested chickens representing eight breeds and absent from all 433 non-crested chickens representing 214 populations. The mutation causes ectopic expression of at least five closely linked *HOXC* genes, including *HOXC10*, in cranial skin of crested chickens. The result is consistent with the interpretation that the crest feathers are caused by an altered body region identity. The upregulated *HOXC* gene expression is expanded to skull tissue of Polish chickens showing a large crest often associated with cerebral hernia, but not in Silkie chickens characterized by a small crest, both homozygous for the duplication. Thus, the 197 bp duplication is required for the development of a large crest and susceptibility to cerebral hernia because only crested chicken show this malformation. However, this mutation is not sufficient to cause herniation because this malformation is not present in breeds with a small crest, like Silkie chickens.

## Introduction

**Figure 1:**
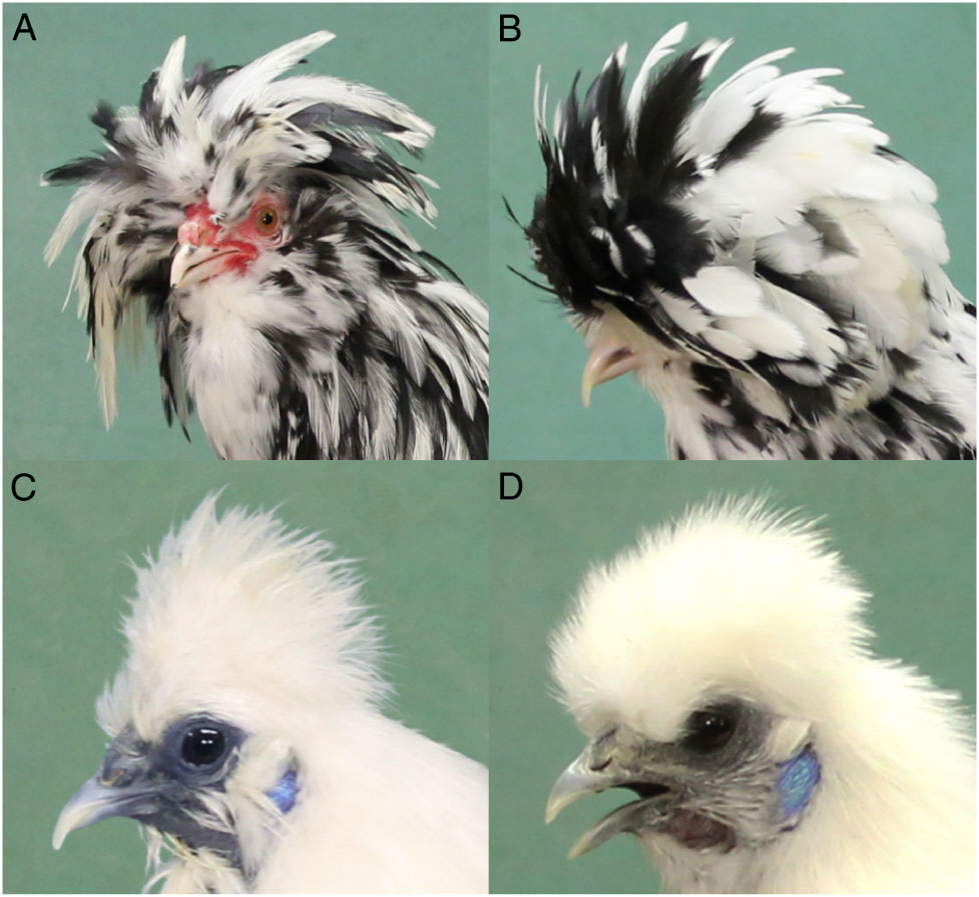
Pictures of adult crested chickens, focusing on the crest phenotypes. (A) Male Houdan, (B) female Houdan, (C) male Silkie, and (D) female Silkie. Photos by Jingyi Li.

Crest (*Cr*) in domestic chicken is a homeotic mutation where the small feathers normally present on the head are replaced by the type of feathers present in dorsal skin (Wang *et al.* 2012). Archeological evidence as well as text by the Roman author Claudius Aelianus show that the crest phenotype appeared during the early evolution of the domestic chickens, at least as early as in the third century AD (Brothwell 1979; Wang *et al.* 2012). A crest-like phenotype is also found in many wild birds, for instance in the crested tit (*Lophophanes cristatus*) and hoopoe (*Upupa epops*). Crest is an attractive trait in fancy chicken breeding, and therefore fixed in certain breeds including Silkie and Houdan (Figure 1). Crest is determined by an autosomal gene variant (*Cr*) showing incomplete dominance; and the crest feathers vary in size from rather small in Silkie to large in other breeds like Crevecoeur, Houdan (Figure 1), Polish, and Sultan (Davenport 1906; Somes 1990). A larger and more voluminous feather crest has been associated with cerebral hernia (Fisher 1934; Frahm and Rehkämper 1998; Yoshimura *et al.* 2012). Already in the 19th century, Darwin (1868) reported that in Polish chickens, the cerebral hemispheres were extruded into the spherical region of the skull and the anterodorsal part of the skull is expanded into a large spherical protuberance. This condition named “cerebral hernia” has also been reported in the Houdan (Davenport 1906), Crevecoeur and Sultan (Shelton 2010) breeds, but not in Silkies (Dunn and Jull 1927). This is consistent with a report by Krautwald (cited by Warren and Hutt 1936) that the size of the crest is directly proportional to the degree of cerebral hernia. Chicken skulls with cerebral hernia have been found at various archeological sites in Western Europe (Great Britain, Austria, Germany, and Hungary) from the Roman to the Post-Medieval period (Gál *et al.* 2010).

Wang *et al.* (2012) mapped *Cr* to the *HOXC* cluster on an unplaced scaffold using the GalGal3 assembly, and documented ectopic expression of *HOXC8* in cranial skin of crested chicken embryos. In wild-type chickens, different Hox genes are expressed in a region-specific way in dorsal skin along the anterior-posterior axis of the chicken embryo and were proposed to control feather identity (Chuong *et al.* 1990). *HOXC8* is expressed in dorsal skin but not in cranial skin during the initial stages of feather follicle formation (Kanzler *et al.* 1997). Hence, it was proposed that ectopic expression of *HOXC8* in cranial skin changes body region identity so that crest feathers resemble dorsal skin feathers (Wang *et al.* 2012). However, it was not possible to identify the causal mutation for crest at the time because of the poor assembly of this genomic region in the GalGal3 assembly (Wang *et al.* 2012). No mutation responsible for cerebral hernia has yet been identified (Verdiglione and Rizzi 2018). It has been proposed that crest and cerebral hernia are determined by the same locus, or two closely linked loci (Davenport 1906; Fisher 1934; Warren and Hutt 1936; Bartels 2003).

In this study, the causal mutation for crest was identified by taking advantage of an improved genome assembly (GalGal6), and by performing identical-by-descent (IBD) mapping using publicly available whole-genome sequencing (WGS) data. We report that the causal mutation for *Crest* is a 197 bp duplication involving an evolutionarily conserved sequence in the intron of *HOXC10.* All crested chickens we have tested carry this mutation. The mutation alters the expression pattern of a cluster of *HOXC* genes on chicken chromosome 33.

## Materials and methods

### Animals and collection of tissue samples

Tissue samples (cranial skin, dorsal skin, ventral skin, skull, and brain) for RNA isolation were collected from three White Crest Black Polish, three Black Silkie and two red junglefowl (RJF) embryos or chicks at developmental stages E10, E13, E17, and D1. One White Leghorn embryo and one White Crest Black Polish were harvested at E13 for hematoxylin & eosin (H&E) staining and *in situ* hybridization. Fertile eggs of Polish chickens were obtained from Ideal Poultry (http://www.idealpoultry.com/, Cameron, TX, USA). Fertile eggs from RJF came from a population kept at Texas A&M University’s Poultry Research Center. Fertile eggs from White Leghorn were procured from Charles River Laboratories, Preston, CT, USA. Procedures for the tissue sample collections followed by the guidelines of Institutional Animal Care and Use Committee at Texas A&M University College of Veterinary Medicine & Biomedical Sciences.

### *In silico*-mapping of PCR primers

A total of 23 markers were used for linkage mapping in the study of Wang *et al.* (2012). In the current study, *in silico* PCR was carried out (https://genome.ucsc.edu/cgi-bin/hgPcr?hgsid=795624545_S0OTcFrLdlKSc9rMXBA5shQNAalK) using the primer sequences used for genotyping these markers, in order to locate them in the current genome assembly, GalGal6.

### Whole-genome sequencing

Publicly available WGS data from 219 individuals or pooled samples were analyzed (Supplementary Table S3). It included 22 samples from crested chickens and 197 samples from non-crested chickens. All Illumina paired-end FASTQ data were aligned to the GalGal6 genome assembly using BWA (version: 0.7.12), sorted with SAMtools (version: 1.6), and variants were called with GATK HaplotypeCaller 3.8 according to Broad Best Practices (Poplin *et al.* 2017). Structural variants were called with Lumpy (version: 0.2.13) (Layer *et al.* 2014). A search for sequence variants within the duplicated region that may not be called by GATK or Lumpy was done by visualizing the alignment files in the integrative genomics viewer (IGV, version: 2.4.3).

### Genotyping

Each of the samples listed in Supplementary Table S4 were genotyped by PCR for the 197 bp duplication, using the following primers: Cr_197_F1: 5′- ACCAAACCGCTTCGATGTGT-3′ and Cr_197_R1: 5ʹ- CGTCCCATTGGCATCACC-3ʹ. PCR assays were conducted following the standard protocol for Platinum™ Green Hot Start PCR Master Mix (2X) (Invitrogen). A standard touch-down protocol was used for the amplification. The amplification generates a 438 or a 241 bp fragment due to the presence/absence of the 197 bp duplication. The PCR products were analyzed by agarose gel electrophoresis. All PCR products with the 438 bp fragment were Sanger sequenced, to genotype the mutations within the duplication, whereas all the PCR products with the 241 bp fragment were genotyped for the SNP via the KASP assay, developed by LGC Genomics (Beverly, MA, USA; www.lgcgenomics.com) (Semagn *et al.* 2014). KASP assays were conducted with a mix of 2.5 μl of KASP V4.0 2X Mastermix (LGC Genomics, Beverly, MA, USA; www.lgcgenomics.com), 1.5 μl PCR-grade water, 1 μl DNA (25 ng/μl), and 0.07 μl of primers mix (12 μM each of allele-specific primer, carrying standard FAM or HEX compatible tails, and 32 μM of allele-flanking primer). Amplifications were carried out on a Bio-Rad CFX384 Touch™ Real-Time PCR Detection System. The PCR amplification protocol began with 94° for 15 min, 10 cycles of 94° for 20 s and 61° (−0.6°/cycle) for 1 min each, followed by 26 cycles of 94° for 20 s and 55° for 1 min each. The protocol ended with an endpoint fluorescence reading after incubation at 37° for 1 min. The readings were analyzed using the Bio-Rad CFX Manager™ Software. Genotyping results were validated by at least two replicates for each sample.

One DNA sample from Houdan was PCR amplified and Sanger sequenced for a 4.1 kb region including the 1.9 kb IBD region, using the primers listed in Supplementary Table S6. Two DNA samples from Ameraucana chickens were PCR amplified and Sanger sequenced using the primers Cr_IBD_F2, Cr_IBD_R2, Cr_IBD_F3, and Cr_IBD_R3, to genotype the four SNPs (Chr33:7,586,870, 7,586,999, 7,587,089, and 7,587,313 bp) within the IBD region.

### TRANSFAC analysis

Predictions of putative transcription factor binding sites were done by the MATCH program in the TRANSFAC database (Kel *et al.* 2003). The vertebrate database was used and only predicted binding sites with a core score of 1.0 were selected for further analysis.

### Quantitative real-time RT-PCR

Total RNA was extracted using Quick-RNA Miniprep Plus Kit (Zymo Research). First-strand cDNA was synthesized using SuperScript™ IV VILO™ Master Mix (Invitrogen). qPCRs were conducted with PowerUp™ SYBR™ Green Master Mix (Applied Biosystems). Total RNA isolated from tissue samples from day 1 at hatch were also reverse-transcribed and analyzed for miR-196a expression using miRCURY LNA™ miRNA PCR Starter Kit (QIAGEN). miRCURY miRNA Assay primers set for hsa-miR-196a-5p (Catalog No.—YP00204386) and hsa-let-7a-5p (Catalog No.—YP00205727) were included in the same kit. The products were detected with Bio-Rad CFX384 Touch™ Real-Time PCR Detection System. Details of qPCR of primers are listed in Supplementary Table S7. Each qPCR assay was carried using three technical replicates.

### *In situ* hybridization

Chicken embryos were harvested, then fixed in 4% paraformaldehyde at 4° overnight. The samples were gradually dehydrated with an ethanol series, embedded in paraffin blocks and cut at 7 mm. The paraffin sections were utilized for hematoxylin and eosin staining and for *in situ* hybridization (ISH). The probes used in ISH were synthesized by PCR reactions. cDNA from E7 chicken skin served as DNA template, and the primers are listed in Supplementary Table S8. The PCR products were purified and cloned into the p-drive vector (Qiagen). The amplicons were sequenced and their fidelity and specificity were verified using NCBI Blast. The plasmids were linearized with *Bam*HI or *Sal*I and transcribed using digoxigenin-labeled nucleotides and SP6 or T7 RNA polymerase to construct probes. The procedures of ISH were as described (Chang *et al.* 2019). Lastly, a counterstain of 0.5% eosin was applied for 15 s. For semi-quantification, the White Leghorn and Polish chicken samples were embedded in the same paraffin block and sectioned.

### Data availability

Data presented in the following supplementary figures and tables are available at figshare.

Supplementary Figure S1: Sequence alignment between *cr^WT^* and *Cr* haplotypes. Supplementary Figure S2: Predicted *MIR196A* binding site in or in the vicinity of *HOXC8* genes in some vertebrate species. Figure S3: Predicted transcription factor binding sites specific to *Cr2*. Supplementary Tables S1 and S2: Marker information for linkage analysis. Supplementary Table S3: Publicly whole genome sequence data used in this study. Supplementary Table S4: DNA samples used for diagnostic tests in this study. Supplementary Table S5: Predicted transcription factor binding sites affected by the duplication. Supplementary Tables S6–S8: Information of primers used in this study.

Supplementary material is available at figshare: https://figshare.com/s/9be524a79413428ac9c2.

## Results

### Identification of the causal mutations

The genetic markers used in the previous mapping study of the *Cr* locus (Wang *et al.* 2012) were assigned to the current GalGal6 assembly using the reported primer sequences. Based on these data, we were able to define the candidate region harboring the *Crest* locus as a 250 kb interval from marker *HOXC-SCF2* (7.57 Mb of chromosome 33) to the distal end of chromosome 33 (7.82 Mb) (Supplementary Tables S1 and S2).

**Figure 2:**
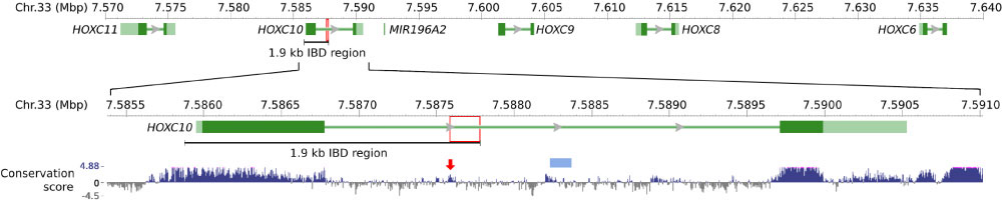
Localization of the causal mutation for the crest phenotype on chicken chromosome 33. The 77 vertebrates basewise PhyloP conservation scores (https://hgdownload.soe.ucsc.edu/goldenPath/galGal6/phastCons77way/) are shown at the bottom. The red boxes indicate the location of the 197 bp duplication causing the crest phenotype. It is partially overlapping a conserved element (red arrow). The blue solid box represents an ATAC-seq merged peak (Foissac *et al.* 2019).

We then analyzed WGS data from 22 samples (18 individuals and 4 pools) representing eight breeds with crest and 197 with the wild-type phenotype (Supplementary Table S3) under the assumption that these crested chickens have inherited the *Cr* mutation from a common ancestor. The analysis of the 250 kb candidate region revealed a 1.9 kb IBD region (Chr33:7,585,881–7,587,784 bp) shared by all crest samples (Figure 2). We found five sequence changes within the IBD region, including four single nucleotide variants (Supplementary Figure S1) and one duplication. Only one of them, a 197 bp tandem duplication of the sequence Chr33:7,587,588–7,587,784 bp was present in all crested but absent from all wild-type chickens. The results provide genetic evidence that this duplication must be the causal mutation for the crest phenotype.

**Figure 3:**
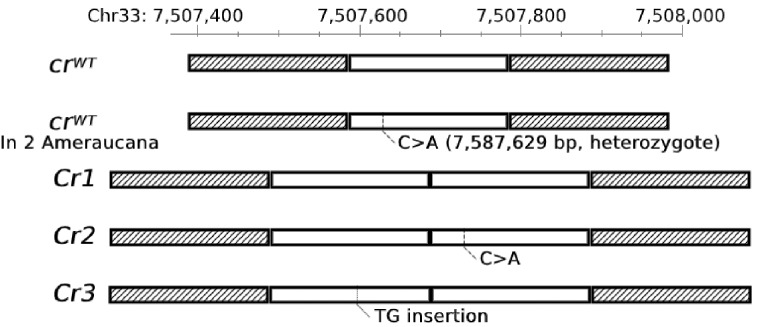
Schematic diagram of five different alleles at the *Crest* locus. Open box indicates the 197 bp sequence, which is tandem duplicated in *Cr1*, *Cr2*, and *Cr3*. Shaded boxes indicate the flanking regions. Dashed vertical lines indicate the same SNP associated with *Cr2* and *cr^WT^* in two Ameraucana samples. Dotted vertical line indicates the 2 bp insertion associated with *Cr3*.

Previous studies indicated that crest and cerebral hernia are determined by the same locus or two closely linked loci (Davenport 1906; Fisher 1934; Warren and Hutt 1936; Bartels 2003). Therefore, within the same 250 kb region defined by linkage analysis of the Crest locus, we also searched for IBD shared among the 13 samples representing breeds (Crested Dutch, Houdan, Polish, and Dutch-Polish) with both an extensive crest and cerebral hernia and the same 1.9 kb IBD region associated with *Cr* was identified. Further analysis revealed a single nucleotide substitution in the 3ʹ copy of the 197 bp duplication (corresponding to nucleotide g.7,587,629C>A on chromosome 33 in the non-duplicated sequence, Figure 3). We designate the allele without the single base change *Cr1* and the one with the single base change *Cr2* (Figure 3). A third Crest-associated allele, designated *Cr3*, was also identified. *Cr3* carries the 197 bp duplication and has a 2 bp insertion in a short tandem repeat (Chr33:7,587,694(TG)_7_>(TG)_8_) in the 5ʹ copy of the duplication (Figure 3). *Cr2/Cr2*, *Cr2/Cr3*, and *Cr3/Cr3* are the only genotypes found in the 13 crested chicken samples with large crest but in none of the six samples representing breeds with a small crest (Silkie and Beijing You), nor in any of the 197 wild-type chickens with WGS data representing 51 breeds (Supplementary Table S3). We also analyzed WGS data for two Appenzeller and one Schijndelaar chicken samples, which grow crest feathers only upward, unlike the large or small crested chickens. Whether they express cerebral hernia is unknown. They were found to also carry *Cr2* or *Cr3* but not *Cr1* or *cr^WT^*. A 4.1 kb region including the 1.9 kb IBD region was Sanger sequenced in Houdan, confirming the WGS finding of the 197 bp duplication and the single base change in the 3ʹ copy.

Diagnostic tests for the *Crest* locus, a combination of fragment analysis of PCR amplicons, Sanger sequencing, and Kompetitive allele-specific PCR (KASP) assay, were carried out using 284 individual samples representing 171 different chicken populations, 29 crested and 142 not. All individuals from the 142 non-crested populations were genotyped as wild-type (*cr^WT^*/*cr^WT^*). The 28 crest purebred populations, are fixed for either *Cr1*, *Cr2* or *Cr3* (Supplementary Table S4); two crested individuals from a cross between New Hampshire (non-crested) and Silkie (small crest) were heterozygous *Cr1*/*cr^WT^*. The genotype data for purebred chickens based on WGS data and the diagnostic tests are summarized in Table 1.

**Table 1:** Summary of *Cr* genotyping results across breeds with or without crest. Data based on whole-genome sequencing or diagnostic tests

Breed	Phenotype	Genotype				
		*Cr1*/* Cr1*	*Cr2*/* Cr2*	*Cr2*/* Cr3*	*Cr3*/* Cr3*	*cr^WT^*/* cr^WT^*
Beijing You	Small crest	1	0	0	0	0
Silkie	Small crest	22	0	0	0	0
Crested Dutch	Large crest	0	1	0	0	0
Crevecoeur	Large crest	0	1	0	0	0
Dutch-Polish	Large crest	0	3	1	4	0
Houdan	Large crest	0	8	0	0	0
Polish	Large crest	0	11	2	1	0
Sultan	Large crest	0	5	0	2	0
Appenzeller	Upward crest	0	4	0	0	0
Schijndelaar	Upward crest	0	1	1	0	0
Other breeds	Non-crested	0	0	0	0	433

The KASP assay for the g.7,587,629C>A SNP was used to genotype all birds from the 142 non-crested populations. Two Ameraucana individuals were found to be heterozygous for this SNP, whereas all other were wild-type. The four single nucleotide changes in the 1.9 kb IBD region associated with the *Crest* haplotype (Supplementary Figure S1) were therefore genotyped and the duplication status was assessed in these two Ameraucana birds. This analysis revealed that they did not carry the 197 bp duplication but they were heterozygous for a haplotype with a sequence identical to one of the copies in *Cr2* with the g.7,587,629C>A substitution (Figure 3). This haplotype most likely evolved from the *Cr2* haplotype, as the first copy of the duplication was lost and left the second copy with the g.7,587,629C>A substitution. This haplotype was not found in any of the 197 samples of non-crested chicken with WGS data. This finding of a putative back mutation further supports the causality of the 197 bp duplication because Ameraucana chickens do not show the crest phenotype.

### Prediction of upstream transcription factors

The duplication is located within the single intron of *HOXC10* and partially overlaps a 133 bp conserved element (mean PhyloP score = 0.43 based on the 77 vertebrates basewise PhyloP conservation score, https://hgdownload.soe.ucsc.edu/goldenPath/galGal6/phastCons77way/) (Figure 2), suggesting its functional importance, possibly as a regulatory element controlling gene expression. TRANSFAC analysis (Kel *et al.* 2003) predicted that the duplication junction creates new transcription factor binding sites for three transcription factors, whereas a large number of putative transcription factor binding sites have been duplicated (Supplementary Table S5). Furthermore, an ATAC-seq merged peak (Foissac *et al.* 2019) was found only about 400 bp downstream of the duplication (Figure 2) in liver and T-cell samples from non-crested White Leghorn chickens, supporting the notion that a regulatory domain is located in this intron.

### Ectopic expression of genes in the *HOXC* cluster in crested chickens

**Figure 4:**
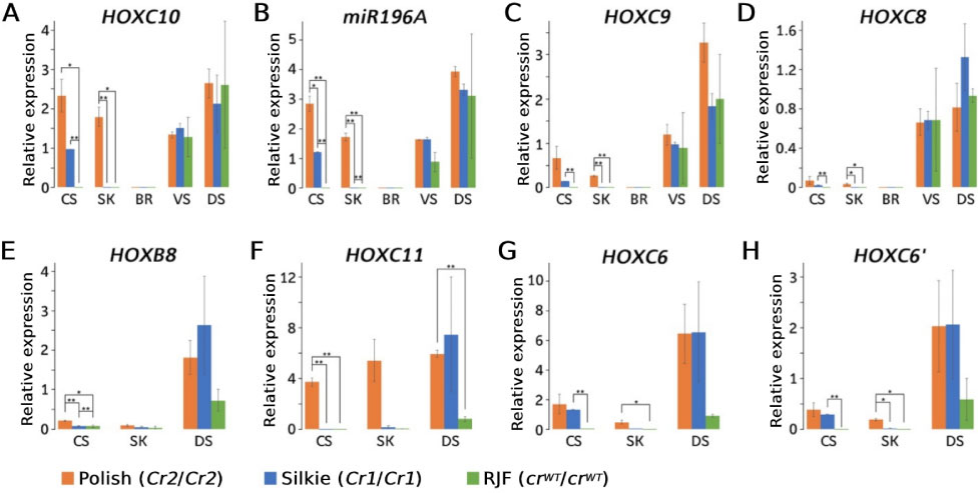
Results of RT-qPCR analysis of *HOXC* genes and *miR196A* in chicken using samples collected at hatch. CS, cranial skin; SK, skull; BR, brain; VS, ventral skin; DS, dorsal skin; RJF, red junglefowl. For each combination of gene and tissue the results are presented in the following order Polish, Silkie, and RJF. For each gene, relative expressions are reported as proportion to the expression level in dorsal skin of one of the RJF. Expression of *miR196A* was normalized against *miR-Let-7a* expression, expressions of other genes were normalized against *GAPDH* expression. Sample size: Polish (*N* = 3), Silkie (*N* = 3), and RJF (*N* = 2). (G) *HOXC6* qPCR primers only amplify the mRNA isoform XM_003643454.3. (H) *HOXC6*’ qPCR primers only amplify the mRNA isoform XM_015300352.2.

The three crest-associated mutations detected in this study are located within the single intron of *HOXC10*, whereas ectopic expression of *HOXC8* was previously reported to be associated with crest (Wang *et al.* 2012). We therefore explored the expression patterns of these genes and those located between them (microRNA *miR196A2* and *HOXC9*, Figure 2) across five tissues (cranial skin, dorsal skin, ventral skin, skull, and brain) collected at hatch (D1) from Silkie (*Cr1/Cr1*), Polish (*Cr2/Cr2*) and red junglefowl (RJF, *cr^WT^*/*cr^WT^*). All four genes showed similar expression patterns: 1. they are not expressed in brain; 2. they are expressed in dorsal and ventral skins with no significant difference between genotypes; 3. they are highly expressed in Polish cranial skin, moderately expressed in Polish skull and Silkie cranial skin, but they are not expressed in Silkie skull, RJF cranial skin, or RJF skull (Figure 4, A–D).

*miR196A* is known to down-regulate *HOXC8* and *HOXB8* by binding to the 3ʹUTR in human (Mueller and Bosserhoff 2011; Hilton *et al.* 2019) and mouse (Mansfield *et al.* 2004; Yekta *et al.* 2004; Mori *et al.* 2012). The sequence of *miR196A*, and the binding site sequences which are approximately 1.2 kb downstream from the 3ʹ-end of the *HOXC8* coding region, are highly conserved in vertebrates including chicken (Yekta *et al.* 2004). However, the putative *miR196A* binding site is not within the 3ʹUTR region of chicken *HOXC8* according to the gene model in GalGal6, NCBI annotation (Supplementary Figure S2). This is consistent with our qPCR data that the expression of *miR196A* followed the same trend as *HOXC8*, which suggests that *miR196A* is not inhibiting the expression of *HOXC8* (Figure 4, B and D). The 3ʹUTR of chicken *HOXB8* is also short so that the putative binding site of *miR196A* is beyond the 3ʹUTR of chicken *HOXB8*. Whether *HOXB8* is down-regulated in crest chickens as a consequence of upregulated expression of this microRNA was investigated by qPCR. The expression of *HOXB8* in cranial skin and skull were lower than that in dorsal skin. However, no significant difference was detected between breeds (Figure 4E). Thus, this suggests that up-regulated expression of *miR196A2* in crested chicken is most likely not relevant for the manifestation of the crest or cerebral hernia phenotypes.

**Figure 5:**
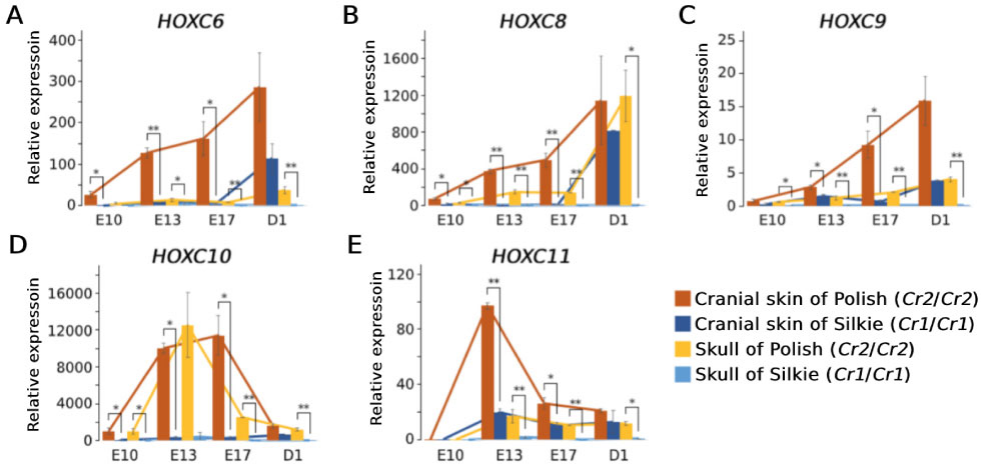
Results of RT-qPCR analysis of *HOXC* genes using cranial skin samples from different developmental stages. E10, E13, and E17 represent embryo stages day 10, 13, and 17, respectively. D1 represents day 1 at hatch. For each gene, relative expressions are reported as proportion to the expression level in the cranial skin of one of the red junglefowl at E13; gene expressions in the red junglefowl samples are not shown as all genes at all stages are barely detected. Gene expression was normalized against *GAPDH* expression. Sample size: Polish (*N* = 3), Silkie (*N* = 3), red junglefowl (*N* = 2).

Further investigations focused on the expression of five *HOXC* genes (*HOXC6*, *HOXC8*, *HOXC9*, *HOXC10*, and *HOXC11*) located at the distal end of chromosome 33 (Figure 2). We isolated mRNA from three tissues, cranial skin, skull, and dorsal skin collected from embryo stages E10, E13, E17, and at hatch. In general, the expression of the different genes at different stages largely follow the same patterns: 1. they are expressed in dorsal skin with no significant difference between breeds; 2. they are highly expressed in Polish cranial skin, moderately expressed in Polish skull and Silkie cranial skin, but they are not expressed in Silkie skull, RJF cranial skin, or RJF skull (Figures 4 and 5). In some tissues, like Polish cranial skin, although they are all expressed through all four stages, the expression levels vary between genes and over developmental stages. Their expression patterns during the embryo development can be summarized as follows: (1) the expression of *HOXC6*, *HOXC8*, and *HOXC9* are increasing from E10 to D1 (Figure 5, A–C); (2) the expression of *HOXC10* and *HOXC11* firstly increase and then decrease, from E10 to D1 (Figure 5, D and E).

**Figure 6:**
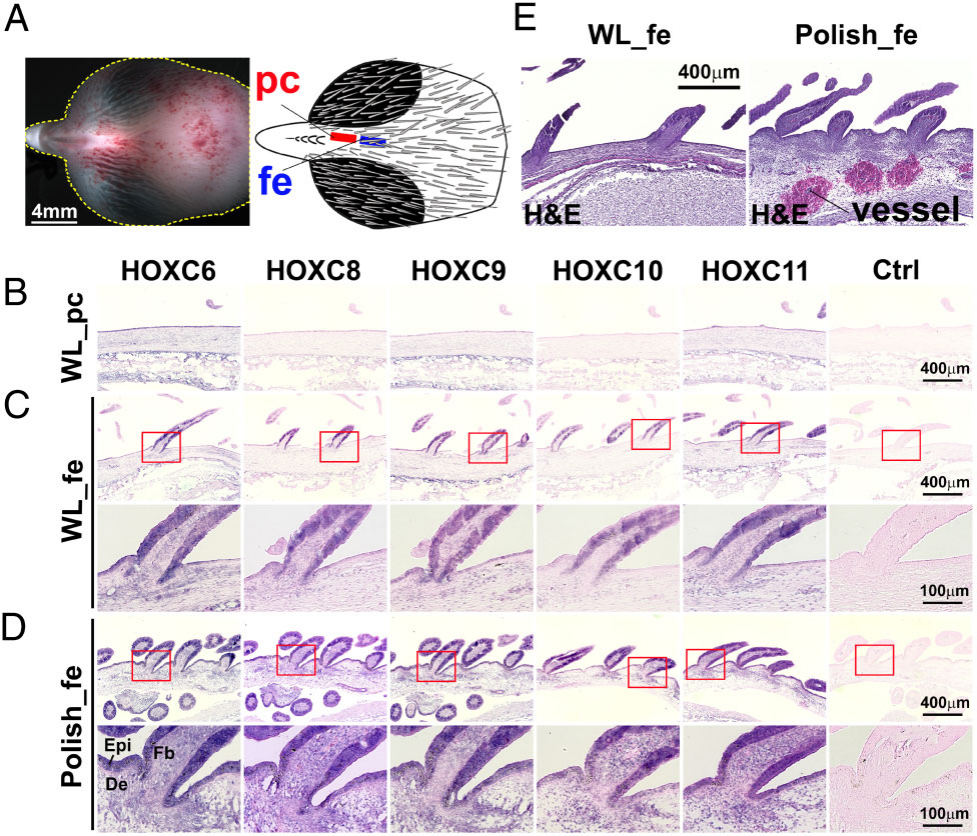
ISH of *HOXC* genes on the scalp skin of E13 chicken embryos. (A) E13 White Leghorn (WL) head showing cranial skin. White dots mark the contour of the head. The cranial skin is composed of the putative comb region (pc) and adjacent feather growing regions (fe) (indicated in red and blue, respectively). (B–D), Section ISH using *HOXC6*, *HOXC8*, *HOXC9*, *HOXC10*, and *HOXC11* probes, respectively; the most right column is the control with no added RNA probe. (B) Sagittal sections of the putative comb region in WL chicken show no signal. (C) Sagittal sections of the feather growing region in WL chicken show staining on bud epidermis but absence in dermis. (D) Polish chicken. High level and diffuse Hox C staining are seen in both epidermis and dermis. (E) H&E staining of the feather growing cranial skin region in WL (left) and in Polish chicken (right). Polish chicken skin shows longer feathers, dense upper dermis and abnormally enlarged blood vessels. De, dermis; Epi, epidermis; Fb, feather bud; fe, feather-growing region; pc, putative comb region; WL, White Leghorn.

RT-PCR was done with whole skin dissected from the indicated region. To see whether the expression of *HOXC* genes are in epidermis or dermis, we then performed *in situ* hybridization using cranial skin from crested Polish chicken and non-crested White Leghorn at E13. In the cranial skin of White Leghorn, there is a putative comb region where no feathers grow. Peripheral to the comb, there are still feather growing regions (Figure 6A). The putative comb region showed no expression of these five *HOXC* genes (Figure 6B). In the adjacent feather growing region of White Leghorn, *HOXC6*, *HOXC8*, *HOXC9*, *HOXC10*, and *HOXC11* genes are expressed at a low level in the feather bud epidermis, but neither in the dermis nor in the interbud epidermis (Figure 6C). In contrast, in Polish chicken, these five *HOXC* genes showed strong expression in whole cranial epidermis, including feather bud and interbud epidermis, and the dermis (Figure 6D). Although *in situ* hybridization data are not very quantitative, we examined these skin specimens on the same slides to provide the best possible estimation of their relative expression levels. In general, wider and higher expression levels of *HOXC* genes are observed in the feather growing region of Polish cranial skin, compared to the expression in the same region of White Leghorn.

We also studied the skin architecture of crested Polish chicken and the feather growing region of non-crested chicken (White Leghorn) using H&E staining. The results revealed that the upper dermis of the Polish chicken skin appears to be thicker and has higher dermal cell density. In the lower dermis, there are abnormally enlarged blood vessels (Figure 6E). Taken together, the results confirm higher expression of all five *HOXC* genes in the Polish cranial skin compared with White Leghorn cranial skin and suggest that this is causing the development of crest feathers.

## Discussion

Crest is a homeotic mutation and here we present genetic evidence that this phenotype is caused by a 197 bp duplication of a conserved element located in the intron of *HOXC10* on chicken chromosome 33. This is based on the identification of a 1.9 kb IBD region present in all crested chickens for which WGS data are available; and within this region the duplication is the only sequence variant unique to crested chicken. A diagnostic test revealed that the duplication was present in all 68 crested chickens representing 11 breeds and absent in 433 non-crested individuals representing 214 populations (Table 1). We identified three different alleles (*Cr1-Cr3*) carrying the duplication. The difference between the alleles are that *Cr2* carries an additional single base change in the 3ʹ copy of the duplication (Chr33: g.7,587,629C>A), whereas *Cr3* has an expansion of a short TG dinucleotide repeat (Chr33: g.7,587,694(TG)_7_>(TG)_8_) in the 5ʹ copy (Figure 3). The *Cr1* allele appears to be fixed in the Asian breeds Silkie and Beijing You, whereas breeds with a European origin, like Houdan and Polish, only carry *Cr2* or *Cr3*. Thus, a likely scenario is that *Cr1* arose in Asian chicken and accumulated additional sequence changes after the introduction to Europe that happened at least as early as about 2,000 years ago (Brothwell 1979; Wang *et al.* 2012). At present it is not possible to judge whether any of the two additional sequence variants present in *Cr2* and *Cr3* is functionally important and may contribute to the development of a larger crest in European crested chicken (Figure 1 and Table 1).

Appenzeller Spitzhaubenhuhn chickens express crest which projects upwards and slightly forwards, unlike that of most other crested breeds consisting of elongated feathers all around the head. Joller *et al.* (2018) found that there is no clear association between markers around or within *HOXC8* with the presence of crest in Appenzeller chickens. Here we show that four Appenzeller chicken from three different populations all carry the 197 bp duplication in *HOXC10* and are homozygous *Cr2/Cr2*, suggesting that the difference between upward crest and large crest, is controlled by other genetic factor(s). Such mutation(s) could be identified by a cross-breeding experiment between Appenzeller and Polish chicken.

The *HOXC* cluster on chicken chromosome 33 contains nine *HOXC* genes, and we explored the expression pattern of five of these, *HOXC6, HOXC8, HOXC9, HOXC10*, and *HOXC11* (Figure 2) using both qPCR and *in situ* hybridization. This analysis revealed a marked upregulated expression of all five in cranial skin from both crested Silkie and crested Polish chicken compared with wild-type controls across different developmental stages (Figures 4–6). However, there was a clear difference in expression pattern in the two types of crested chicken as Polish chicken, showing a large crest, had a more pronounced upregulated *HOXC* expression that also affected the skull compared with Silkie chickens carrying a small crest (Figure 1). Wang *et al.* (2012) previously reported ectopic expression of *HOXC8* but not of *HOXC12* or *HOXC13* in Silkie cranial skin; the two latter genes were therefore not included in the present study. Our finding that the 197 bp duplication causing crest, overlapping a conserved sequence, affects the expression of five *HOXC* genes in chicken implies that the duplication alters the chromatin structure in the region. This may be caused by the creation or duplication of one specific transcription factor binding site or the combined effect of the duplication of multiple binding sites. A reasonable interpretation is that the 197 bp duplication includes a *HOXC* enhancer element that becomes substantially stronger by the duplication, as previously shown using transgenic zebrafish experiments for the duplication causing Greying with age in horses (Sundström *et al.* 2012). Wang *et al.* (2012) proposed that ectopic expression of *HOXC8* in cranial skin changes body region identity and that the crest feathers resemble feathers generated from dorsal skin where the *HOXC* genes are expressed in wild-type chickens. The present study fully supports the interpretation of an altered body region identity but shows that the mechanism may involve ectopic expression of a whole cluster of *HOXC* genes.

The enlarged blood vessels observed in the lower dermis, and thickened upper dermis of Polish cranial skin, which matches previous observation (cited by Yoshimura *et al.* 2012) further suggest that the effects of ectopic *HOXC* gene expressions are expanded into dermis, and thus likely affect even deeper tissues, and may cause the cerebral hernia observed in crested chickens. The 197 bp duplication in *HOXC10* is required but not sufficient in causing cerebral hernia because this malformation is exclusively found in birds carrying the *Cr2* or *Cr3* alleles and with a large crest, like Polish chicken, but not in those with a small crest, like Silkie, carrying the *Cr1* allele. At present, it is still an open question whether a large crest and the associated cerebral hernia are caused by cis-acting mutation(s) affecting *HOXC* expression or mutation(s) elsewhere in the genome affecting the growth of crest feathers and leading to malformation of the skull. Previous cross-breeding experiments suggest that cerebral hernia is caused by a pleiotropic effect of the *Crest* mutation or a closely linked recessive mutation (Yoshimura *et al.* 2012). We noted a clear difference in *HOXC* expression between Silkie and Polish chicken, as the latter had ectopic expression of *HOXC* genes not only in cranial skin but also in skull tissue (Figure 4). In addition, the peak of expression for *HOXC10* and *HOXC11* in Polish skull occurs during embryo stage E13 (Figure 5, D and E), whereas the expression of the other three genes increases over time (Figure 5, A–C). It is possible that craniofacial (skull) development, which is more critical at early embryonic stages (Ashique *et al.* 2002; Holleville *et al.* 2003), is more affected by the upregulated expression of *HOXC10* and/or *HOXC11*. At the later stages when down feathers start to grow (Yu *et al.* 2004), the other three genes (*HOXC6, HOXC8, HOXC9*) may contribute more to the crest phenotype. The study of *Koa* mouse suggests that each ectopically expressed *HOXC* gene may contribute additively to hair growth on the ear of mouse (Yu *et al.* 2018). It is an open question whether *HOXC* genes in chicken also have similar additive effects on feather growth.

The single base change present in the *Cr2* allele (Chr33: g.7,587,629C>A) is a candidate cis-acting causal mutation for large crest and susceptibility to cerebral hernia because we noted that this mutation results in the creation of predicted binding sites for several transcription factors that have been reported to have a role in hair or skull development: Msx-2 (Florisson *et al.* 2013; Simon *et al.* 2014; Hughes *et al.* 2018), HOXB13 (Kömüves *et al.* 2003), Dlx-5 (Depew *et al.* 1999; Robledo *et al.* 2002; Holleville *et al.* 2007), Dlx-3 (Hwang *et al.* 2008; Li *et al.* 2015; Whitehouse *et al.* 2019) (Supplementary Figure S3). The expansion of the short TG dinucleotide present in the *Cr3* allele (Chr33:7,587,694(TG)_7_>(TG)_8_), is less likely to be functionally important. However, the presence of the *Cr2* and *Cr3* alleles in breeds showing large crests and *Cr1* in breeds with a small crest (Table 1) does not provide evidence for a possible causal relationship because the difference occurs between breeds that show many other genetic differences elsewhere in the genome, in particular in this case because *Cr1* occurs in breeds with a Chinese origin whereas *Cr2* and *Cr3* occur in breeds with a European origin. The inheritance of *Crest* in different types of chicken has been studied for more than a century (Davenport 1906; Dunn and Landauer 1930; Fisher 1934; Warren and Hutt 1936; Bartels 2003; Yoshimura *et al.* 2012) since noted by Darwin (Darwin 1868). Now we have identified the causal mutation of *Crest*, a cross-breeding experiment between breeds carrying *Cr1* and *Cr2* or *Cr3* would reveal whether the latter two but not the former is associated with a larger crest and predispose to cerebral hernia. Furthermore, it would be of considerable interest to use gene editing to both confirm causality of the 197 bp duplication and test the possible functional significance of the sequence variants associated with *Cr2* and *Cr3*, but such experiments are still a major undertaking in chicken.

Many spectacular bird plumages such as the crest of the hoopoe (*Upupa epops*), the ornamental feathers of the peacock, the ruff (Lamichhaney *et al.* 2016) and birds of paradise (Gregory 2020) involve feathers of unusual shape and size. Genetic changes in the *HOXC* genes and other transcription factors regulating feather development are top candidate genes underlying this beauty of nature.
