# Corrigendum to “The crest phenotype in domestic chicken is caused by a 195 bp duplication in the intron of *HOXC10*”

**DOI:** 10.1093/g3journal/jkab425

**Published:** 2022-01-04

**Authors:** Jingyi Li, Mi-Ok Lee, Brian W Davis, Ping Wu, Shu-Man Hsieh Li, Cheng-Ming Chuong, Leif Andersson

*G3: Genes|Genomes|Genetics*, 2021. https://doi.org/1093/g3journal/jkaa048

The discovery that a duplication in the intron of *HOXC10* is completely associated with the presence of the crest phenotype in chicken has been confirmed by another group (Zhiwei Liu, Jinyu Chu, Pei Li, Qianqian Zhao, Shijun Li, and Chunyan Mou, personal communication). They noticed a mistake in the calculation of the size of the duplication. The correct size is 195 base pairs not 197 base pairs as indicated before, and the correct genome coordinates for the duplicated sequence in the Galgal6 assembly is position 7,587,588-7,587,782 on chromosome 33.

